# A novel fusion protein consisting of anti-ANGPTL3 antibody and interleukin-22 ameliorates diabetic nephropathy in mice

**DOI:** 10.3389/fimmu.2022.1011442

**Published:** 2022-12-05

**Authors:** Qianqian Ma, Xiaozhi Hu, Fangyu Liu, Zhonglian Cao, Lei Han, Kaicheng Zhou, Yu Bai, Yuting Zhang, Yanyang Nan, Qianying Lv, Jia Rao, Tao Wu, Xue Yang, Haidong He, Dianwen Ju, Hong Xu

**Affiliations:** ^1^ Children’s Hospital of Fudan University, National Children’s Medical Center, Shanghai, China; ^2^ Department of Biological Medicines & Shanghai Engineering Research Center of Immunotherapeutics, School of Pharmacy, Fudan University, Shanghai, China; ^3^ Department of Neurology, The First Affiliated Hospital of Chongqing Medical University, Chongqing, China; ^4^ Department of Nephrology, Minhang Hospital, Fudan University, Shanghai, China

**Keywords:** angiopoietin-like protein 3, interleukin-22, fusion protein, diabetic nephropathy DN, diabetic nephropathy, podocyte injury, inflammatory response

## Abstract

**Introduction:**

The pathogenic mechanisms of diabetic nephropathy (DN) include podocyte injury, inflammatory responses and metabolic disorders. Although the antagonism of Angiopoietin-like protein 3 (ANGPTL3) can alleviate proteinuria symptoms by inhibiting the activation of integrin αvβ3 on the surface of podocytes, it can not impede other pathological processes, such as inflammatory responses and metabolic dysfunction of glucolipid. Interleukin-22 (IL-22) is considered to be a pivotal molecule involved in suppressing inflammatory responses, initiating regenerative repair, and regulating glucolipid metabolism.

**Methods:**

Genes encoding the mIL22IgG2aFc and two chains of anti-ANGPTL3 antibody and bifunctional protein were synthesized. Then, the DN mice were treated with intraperitoneal injection of normal saline, anti-ANGPTL3 (20 mg/kg), mIL22Fc (12 mg/kg) or anti-ANGPTL3 /IL22 (25.3 mg/kg) and irrigation of positive drug losartan (20mg/kg/d) twice a week for 8 weeks.

**Results:**

In this research, a novel bifunctional fusion protein (anti-ANGPTL3/IL22) formed by the fusion of IL-22 with the C-terminus of anti-ANGPTL3 antibody exhibited favorable stability and maintained the biological activity of anti-ANGPTL3 and IL-22, respectively. The fusion protein showed a more pronounced attenuation of proteinuria and improved dysfunction of glucolipid metabolism compared with mIL22Fc or anti-ANGPTL3. Our results also indicated that anti-ANGPTL3/IL22 intervention significantly alleviated renal fibrosis *via* inhibiting the expression of the inflammatory response-related protein nuclear factor kappa light-chain enhancer of activated B cells (NF-κB) p65 and NOD-like receptor family pyrin domain-containing protein 3 (NLRP3) inflammasome. Moreover, transcriptome analysis revealed the downregulation of signaling pathways associated with injury and dysfunction of the renal parenchymal cell indicating the possible protective mechanisms of anti-ANGPTL3/IL22 in DN.

**Conclusion:**

Collectively, anti-ANGPTL3/IL22 bifunctional fusion protein can be a promising novel therapeutic strategy for DN by reducing podocyte injury, ameliorating inflammatory response, and enhancing renal tissue recovery.

## Introduction

Diabetic nephropathy (DN) is a frequent and severe complication of diabetes mellitus (1). It has been described as a significant medical challenge with persistent albuminuria and progressive decline in renal function, eventually inducing chronic renal failure (CRF) and requiring renal replacement therapy (2, 3). DN is attributable to multiple factors such as podocyte injury, abnormal glucose and lipid metabolism, and chronic inflammation. Several available therapeutic interventions, including the correction of hyperglycemia, glucolipid metabolism and lifestyles, have been proven insufficient to prevent the onset of DN and its progression to ESRD (4, 5). Thereby, there is an imperative requirement to discover new therapeutic approaches for DN.

Albuminuria is considered an early predictor of DN progression and is usually relevant to the development of ESRD (6, 7). Evolving evidence suggests that podocyte injury is the primary cause of microalbuminuria observed in early DN (8). Thus, alleviating proteinuria and protecting podocytes are necessary for delaying the progression of DN. Angiopoietin-like protein 3 (ANGPTL3) is a multifunctional secretory protein mainly expressed in the liver. Its coiled-coil domain (CCD) is a key domain that regulates lipid metabolism, and the fibrinogen-like domain (FLD) binds to the integrin αvβ3 receptor and involves in angiogenesis (9). Multiple studies showed that serum ANGPTL3 levels were significantly upregulated in DN patients, positively correlated with urinary microalbumin to creatinine ratio levels, while negatively correlated with GFR (10, 11). In our preliminary studies, a dramatic increase in ANGPTL3 was observed in renal podocytes of patients with nephrotic syndrome (NS) (12–14). Adriamycin-induced podocyte damage and proteinuria were significantly alleviated in *Angptl3^-/-^* mice compared to wild-type mice (15). In addition, we demonstrated that ANGPTL3-FLD played a dominant role in loss of podocyte adhesion and F-actin rearrangement (16, 17). Subsequently, a monoclonal antibody against ANGPTL3-FLD (anti-ANGPTL3) was generated and competitively blocked the binding of ANGPTL3 to integrin αvβ3, thereby inhibiting podocyte damage and attenuating proteinuria. We speculated that restoration of podocyte function and reduction of proteinuria *via* inhibition of ANGPTL3 might be able to improve renal dysfunction in DN.

Several studies have shown that inflammation is the cardinal pathogenic mechanism of DN (18–20). Pro-inflammatory cytokines and chemokines induce changes in glomerular endothelial cell permeability, proliferation of thylakoid cells and increased expression of fibronectin (21). These cytokines are crucial mediators of renal injury due to their ability to promote the migration of monocytes, neutrophils, and lymphocytes (22, 23). The established treatment strategies, such as regulating blood glucose and blood pressure, cannot eliminate the excessive inflammatory response (24). Therefore, therapeutic strategies based on anti-inflammatory effects may translate into promising approaches for the treatment of DN. Interleukin-22 (IL-22) is derived from activated immune cells such as innate lymphoid cells and T lymphocytes, and is primarily involved in epithelial cell functions, including maintaining tissue repair and promoting regeneration (25–27). IL-22 was reported to significantly attenuate acetaminophen- or high-fat diet-induced liver fibrosis by inhibiting the hepatic inflammatory response (28, 29). Our previous research also demonstrated that IL-22 could reduce the inflammatory responses through ameliorating mitochondrial dysfunction and inhibiting the activation of the NOD-like receptor family pyrin domain-containing protein 3 (NLRP3) inflammasome (30, 31). We have found that IL-22 could also alleviate the abnormal lipid metabolism, reduce lipotoxicity in renal cells, attenuate insulin resistance, and ultimately improve the prognosis of DN (32). Therefore, we hypothesized that combining anti-ANGPTL3 and IL-22 could simultaneously reduce proteinuria, mitigate the inflammatory response, and regulate metabolism homeostasis to achieve better DN treatment outcomes.

In our present study, we constructed a novel fusion protein by linking IL-22 to the C-terminus of a mouse anti-ANGPTL3 antibody heavy chain. The fusion protein anti-ANGPTL3/IL22 showed ideal stability and had the capacity to treat DN through alleviating podocyte damage, suppressing inflammation and promoting tissue regeneration. Anti-ANGPTL3 in combination with IL-22 offers a new promising therapeutic strategy to prevent and halt the progression of DN.

## Methods and materials

### Preparation of anti-ANGPTL3 monoclonal antibody, mIL22Fc and anti-ANGPTL3/IL22 bifunctional protein

Genes encoding the mIL22IgG2aFc and two chains of anti-ANGPTL3 antibody and bifunctional protein were synthesized and cloned intothe pTT5 vector using the BamHI/EcoRI or BamHI/HindIII, (New England Biolabs Inc., Ipswich, MA, USA) restriction enzymes. Then the expression vectors were manufactured into ExpiCHO cells (Thermo Fisher Scientific) and the cell supernatants were collected 10 days after transfection. The anti-ANGPTL3 antibody, mIL22IgG2aFc and bifunctional protein were obtained using protein A affinity chromatography.

### SDS-PAGE

SDS-PAGE analysis under reducing conditions was accomplished on 10% Tris-glycine gels. The gels were colored with Coomassie brilliant blue (Invitrogen)and decolorized with a decolorization solution consisting of 10% methanol and acetic acid.

### Thermal stability and SEC-HPLC analysis

Nano Temper PR.48 (Nano Temper Scientific, Germany) was utilized to measure the thermal stability over the temperature range of 20°C to 95°C. All samples were diluted in PBS buffer. The data were fitted to the image and the Tm1 value was considered to be the melting point.

The monomer purity of samples was evaluated using Agilent 1260 Infinity II SFC system. PBS flowed through TOSOH TSKgel G3000WXL column (7.8 mm × 30 cm, 5 μm) at 1.0 ml/min and 100 μgsamoles were calculated by UV detection at 280 nm for 30 minutes.

### Surface plasmon resonance

The affinity determination of anti-ANGPTL3 antibody and anti-ANGPTL3/IL22 fusion protein with mouse or human ANGPTL3 was analyzed by SPR using the Biacore T200 system (GE Healthcare, USA). Firstly, we respectively immobilized human and mouse ANGPTL3 on a CM5 chip (GE Healthcare, USA) at approximately 60 RU by regulating the capture time. Next, anti-ANGPTL3 antibody and anti-ANGPTL3/IL22 fusion protein were diluted by EP buffer and then through the chip for binding at 180s and for dissociation for 480 s. After each round, pH 2.5 Glycine-HCl buffer was used for regeneration. The affinity (KD) was measured as the ratio of the dissociation constant (Kd) to the binding constant (Ka).

### Cell culture

MPC5 cells and mPTC cells were purchased from FuHeng Biology (Shanghai, China). MPC5 were cultured in DMEM, while mPTC were cultured in DMEM/F12 medium (5% CO2, 37°C). DMEM containing 10% FBS, 1% streptomycin and 1% penicillin (Beyotime Biotechnology) was used to culture all cells.

ExpiCHO-S cells (Thermo Fisher Scientific, USA) were cultured in ExpiCHO expression medium with 120 rpm shaking (8% CO2, 37°C) and were transferred to 32°C the day after plasmid transfection. Countstar IC 1000 cell counter was used to assess cell density and viability.

### *In vitro* assay of anti-ANGPTL3/IL22 activity

To assess the biological activity of anti-ANGPTL3/IL22, 1×10^6^ cell/ml mouse glomerular podocytes (MPC5) were inoculated overnight in 6-well plates and induced by high glucose (30 mM). Then cells were incubated with anti-ANGPTL3 (100 ng/ml), anti-ANGPTL3/IL22 (126 ng/ml), and mIL22Fc (90 ng/ml) for 1-2 hours, and subsequently, 30 mM high sugar solution was added for damage intervention for 4-6 hours. Finally, PSI domain-specific antibody AP5 was applied to detect whether anti-ANGPTL3/IL22 could compete for binding to ANGPTL3 to block exposure of PSI epitopes.

To determine the IL-22 activity of anti-ANGPTL3/IL22, mouse proximal renal tubular epithelial cells (mPTC) were inoculated overnight and then were intervened with 20 mM anti-ANGPTL3/IL22 for 2 h, 4 h and 6 h. The supernatant of the lysate was harvested after centrifugation and used for western blot assay.

### Animal models

*db/m* and *db/db* mice (male, 5-6 weeks old) were gained from Changzhou Cavins Animal Experiment Co.Ltd. (Jiangsu, China) and kept under specific pathogen-free (SPF) condition. The *db/m* mice were offered exclusively with chow diet as a control group, while a high-fat diet (40% fat content) was chosen to feed *db/db* mice for 4-5 weeks as a model group. The mouse model of DN was successfully established at about 4 weeks, then treated with intraperitoneal injection of normal saline, anti-ANGPTL3 (20 mg/kg), mIL22Fc (12 mg/kg) or anti-ANGPTL3/IL22 (25.3 mg/kg) and irrigation of positive drug losartan (20mg/kg/d) twice a week for 8 weeks. All animal experiments were approved by the ethics committee of School of Pharmacy, Fudan University.

Blood specimens were collected by capillary tubes through the inner canthus of the eye, and urine was collected in 24-hour mouse metabolic cages. The serum levels of creatinine, urea nitrogen, total cholesterol (T-CHO), triglycerides, low-density lipoprotein (LDL), high-density lipoprotein (HDL), blood glucose, urinary creatinine and 24-hour urinary protein were detected by relevant assay kits (Nanjing Jiancheng Company, Nanjing, China). Urinary microalbumin, serum tumor necrosis factor-α (TNF-α), IL-6 and IL-1β were measured by ELISA kit (Nanjing Jiancheng).

### Histopathology

The kidneys were cut longitudinally, fixed overnight in 4% paraformaldehyde solution, followed by paraffin embedding, and cut into 4-μm-thick slices. The morphological changes were observed using hematoxylin and eosin (H&E), periodontal acid (PAS) and Masson trichrome (Masson) staining. Then, the percentage of positive areas was quantified and analyzed using ImageJ software.

### Electron microscopy analysis

Appropriately-sized kidney tissues were collected in electron microscope (EM) fixative overnight at 4°C. Then electron microscopic observation was performed at 60 kV. Kidney tissues of 3-5 mice in each group were taken to observe the changes in podocyte ultrastructure and glomerular basement membrane thickness.

### Immunofluorescence and immunohistochemical detection

Immunofluorescence staining of MPC5 was performed using AP5 (kerafast, USA, 1:100) and Vimentin (servicebio, China, 1:1500) and α-SMA (servicebio, 1:100) were stained to observe the expression of fibrosis-associated proteins, while the nuclei were labeled with Hoechst 33342.

As for immunohistochemical of kidney tissues, the NLRP3 antibody (Servicebio, 1:1000) and NF-κB p65 antibody (Servicebio, 1:100) were applied for inflammation analysis, and the WT1 antibody (Servicebio, 1:200) and nephrin antibody (Servicebio, 1:200) were used for podocytes protecting effect analysis of anti-ANGPTL3/IL22 analysis. The nuclei were labeled with Hoechst 33342. Finally, images taken were statistically analyzed with ImageJ software.

### Western blot analysis

Total proteins of kidney tissue were collected by homogenizing and quantified with a BCA kit (Beyotime, China). The antibodies for p-STAT3 (CST, USA, 1:1000) was used for Western blot analysis. We then used enhanced chemiluminescent substrates to visualize the target proteins and perform semi-quantitative analysis.

### mRNA sequencing and bioinformatics analysis

Total RNA was extracted from renal tissues and randomly fragmented into small fragments after enrichment by Oligo dT magnetic beads. Double-stranded cDNA was purified using AMPure XP beads and then modified with End Repair Mix to add poly A tails and sequencing connectors. Subsequently, it was converted to single-stranded DNA by USER enzyme, and PCR amplification was performed for 15 cycles to finally form a cDNA library. Finally, PCR clustering was performed on the cBot instrument, and sequencing was completed using the 2*151 mode.

### Statistical analysis

All data are presented as mean ± SEM. Multiple group comparisons were assessed by unpaired Student’s t-test or one-way analysis of variance (ANOVA) followed by Bonferroni *post hoc* test. * *P* < 0.05, **P < 0.01 or ***P < 0.001 was considered as statistically significant difference.

## Results

### Expression and preparation of anti-ANGPTL3/IL22 fusion protein

**Figure 1:**
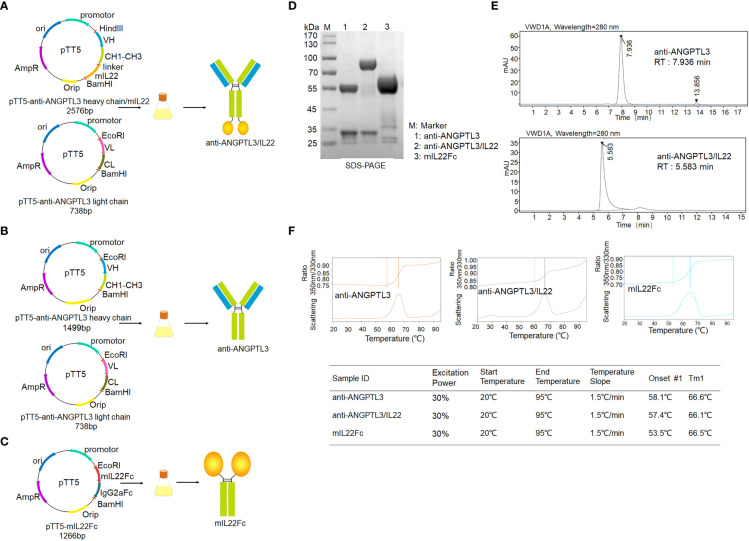
Expression and purification of anti-ANGPTL3/IL22 fusion protein. **(A)** Schematic representation of the preparation and purification of anti-ANGPTL3/IL22 fusion protein. Mouse IL22 was fused to the C-terminus of the anti-ANGPTL3 heavy chain *via* a flexible linker (linker sequence: GGGGSGGGGSGGGGS). **(B)** Schematic representation of the preparation and purification of anti-ANGPTL3. **(C)** Schematic representation of the preparation and purification of mIL22Fc (Mouse IL22 and mouse IgG2a Fc fusion protein). **(D)** SDS-PAGE analysis of anti-ANGPTL3 (lane 1), anti-ANGPTL3/IL22 (lane 2) and mIL22Fc (lane 3) under reducing condition (anti-ANGPTL3: approximately 150 KD, IL22Fc: approximately 90 KD, anti-ANGPTL3/IL22: approximately 190 KD). **(E)** SEC-HPLC analysis of anti-ANGPTL3 and anti-ANGPTL3/IL22 fusion protein (RT, retention time). **(F)** Thermal stability analysis of anti-ANGPTL3, anti-ANGPTL3/IL22 and mIL22Fc.

The gene encoding IL-22 was linked to the C-terminus of the mouse anti-ANGPTL3 heavy chain *via* a flexible linker (Gly4Ser)3 to construct the expression plasmid of the anti-ANGPTL3/mIL22 fusion protein. Then it was co-transfected into CHO-S cells together with the expression plasmid of anti-ANGPTL3 antibody light chain for fusion protein expression (**Figures 1A–C**). SDS-PAGE under reducing conditions and SEC-HPLC results showed the expected molecular weight bands and high purity of anti-ANGPTL3, mIL22Fc and anti-ANGPTL3/IL22 fusion protein (**Figures 1D, E**). The Tm1 values of anti-ANGPTL3 and anti-ANGPTL3/IL22 fusion protein were 66.5°C and 66.6°C, respectively, indicating that the fusion protein had the same favorable thermal stability as the monoclonal antibody (**Figure 1F**).

### Validation of the bioactivity of anti-ANGPTL3/IL22 *in vitro*

**Figure 2:**
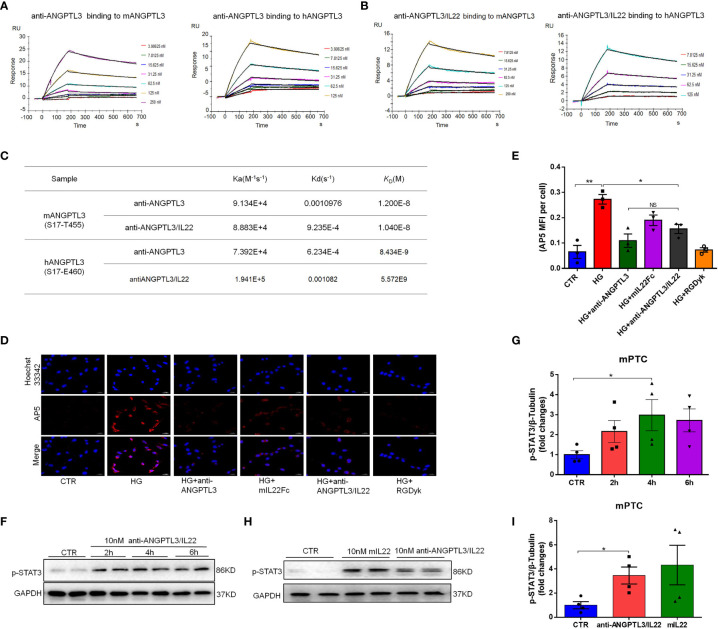
Affinity of anti-ANGPTL3 and anti-ANGPTL3/IL22 for ANGPTL3 and the validation of *in vitro* activity. **(A)** Affinity of anti-ANGPTL3 for mouse (S17-T455) and human (S17-E460) ANGPTL3 measured by surface plasmon resonance (SPR). **(B)** Affinity of anti-ANGPTL3/IL22 for mouse and human ANGPTL3 measured by SPR. **(C)** Affinity summaries of anti-ANGPTL3 antibody and anti-ANGPTL3/IL22 fusion protein suggested that the functional integrity of anti-ANGPTL3 was preserved. **(D)** Mouse podocytes (MPC5) were first incubated with anti-ANGPTL3, mIL22Fc, anti-ANGPTL3/IL22 and FGDyk for 1 hour, followed by the addition of high concentration glucose (30 mmol/L) for 6 hours. Then, the cells were stained with AP5 and Hoechst 33342 to assess intracellular exposed PSI epitopes. (Scale bar: 20 μm). **(E)** Statistical analysis of AP5 levels by immunofluorescence staining (n = 4). **(F)** After mouse proximal tubular epithelial cells (mPTC) were incubated overnight, 10 nM anti-ANGPTL3/IL22-induced phosphorylation of STAT3 (p-STAT3) was analyzed by Western blot at different times. **(G)** Densitometric values of p-STAT3 expression after 2 h, 4 h and 6h. (n = 4). **(H)** Western blot analysis of p-STAT3 induced by 10 nM mIL-22Fc or anti-ANGPTL3/IL22 in mPTC for 6 hours. **(I)** Densitometric values of p-STAT3 expression after 6 hours of intervention. (n = 4). *P < 0.05; **P < 0.01; NS, no significance.

SPR was utilized to investigate the binding affinity of anti-ANGPTL3/mIL22 to ANGPTL3. The results demonstrated that anti-ANGPTL3/IL22 had semblable and favorable affinity to anti-ANGPTL3 antibody regardless of binding to mouse-derived or human-derived ANGPTL3 (**Figures 2A–C**). Meanwhile, anti-ANGPTL3/IL22 could inhibit the exposure of PSI epitope by blocking the binding of integrin ανβ3 to ANGPTL3, which had the same efficacy as anti-ANGPTL3 on the MPC5 cells in the presence of high glucose intervention (**Figures 2D, E**). Besides, immunoblots analysis confirmed that both anti-ANGPTL3/IL22 and mIL22Fc could activate STAT3 phosphorylation in mPTC in a time-dependent manner (**Figures 2F–I**). To observe the safety of the long-term treatment, histological examinations were performed on the major organs after 8-week administration of anti-ANGPTL3/IL22, anti-ANGPTL3 or mIL22Fc, and no significant pathological damage was found. (**Figure S3**). Together, the anti-ANGPTL3/IL22 was successfully constructed and maintained the dual biological effect of anti-ANGPTL3 and IL-22.

### Anti-ANGPTL3/IL22 treatment ameliorated kidney dysfunction, hyperglycemia and dyslipidemia in DN mice

**Figure 3:**
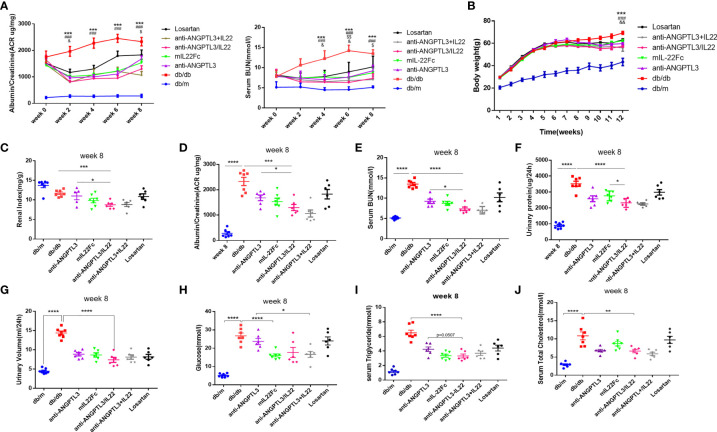
Efficacy in improving renal function of anti-ANGPTL3/IL22 fusion protein in DN mice. **(A)** The tendency of ACR and serum BUN during eight weeks of drug administration. **(B)** Quantitative analysis of body weight trends. * and # respectively represent the Student’s t-test of *db/db* group and anti-ANGPTL3/IL22 treatment compared with *db/m* group at twelve week. $ and & respectively represent the Student’s t-test of anti-ANGPTL3 and mIL22Fc treatment compared with anti-ANGPTL3/IL22 group. **(C)** Renal index (kidney weight/body weight) was measured to reveal the degree of kidney injury. **(D-G)** Trends of ACR, serum BUN, 24-hour urine volume and urine protein in *db/db* mice after 8 weeks of treatment with anti-ANGPTL3/IL22. **(H-J)** Measurements of blood glucose, serum triglyceride and serum T-CHO after the 8 weeks of anti-ANGPTL3/IL22 administration. *P < 0.05; **P < 0.01; ***P < 0.001; ****P < 0.0001. & *P* < 0.05; && *P* < 0.01. $ *P* < 0.05; $$ *P*< 0.01.### *P* < 0.001.

To investigate the effects of anti-ANGPTL3/IL22 on DN, a mouse model of diabete-induced kidney injury was successfully established after feeding *db/db* mice with high-fat chow for 4-5 weeks (**Figures S1A–D**). Mice were given the fusion protein, combinational drugs or the corresponding control drugs for 8 weeks. The results showed that the body weight, renal index and urinary ACR (ratio of kidney weight to body weight, mg/g) were significantly reduced after anti-ANGPTL3/IL22 administration, which showed better improvement than anti-ANGPTL3 or mIL22Fc treatment alone, while the losartan group exhibited only a slight improvement (**Figures 3B, C**, **S2A**). Besides, anti-ANGPTL3/IL22 significantly decreased the serum urea nitrogen (BUN), 24-hour urine volume, and 24-hour urine protein levels, indicating the protective effects on renal function (**Figures 3A, D**–**G**, **S2B–D**).

Serum glucose values were measured to assess the effect of anti-ANGPTL3/IL22 fusion protein treatment on hyperglycemia. The results showed that serum glucose levels were significantly higher in the DN group compared with the *db/m* group (P<0.0001) (**Figure 3H**). Previous studies have reported the efficacy of IL-22 to regulate glucose metabolism by improving insulin resistance (33). In our study, the same hypoglycemic effect was observed in the anti-ANGPTL3/IL22 and mIL22Fc treatment groups. Additionally, elevated levels of triglyceride and total cholesterol in *db/db* mice were mitigated after the pharmacological interventions (**Figure 3I, J**). In particular, the application of anti-ANGPTL3/IL22 resulted in a significant decrease compared with anti-ANGPTL3 or mIL22Fc alone. The trends of changes in blood glucose and lipid levels during 8 weeks of administration are presented in the supplemental figure (**Figures S2E–G**). Both anti-ANGPTL3/IL22 and co-administration significantly ameliorated kidney dysfunction (**Figure 3**).

### Anti-ANGPTL3/IL22 treatment ameliorated histologic changes in DN

**Figure 4:**
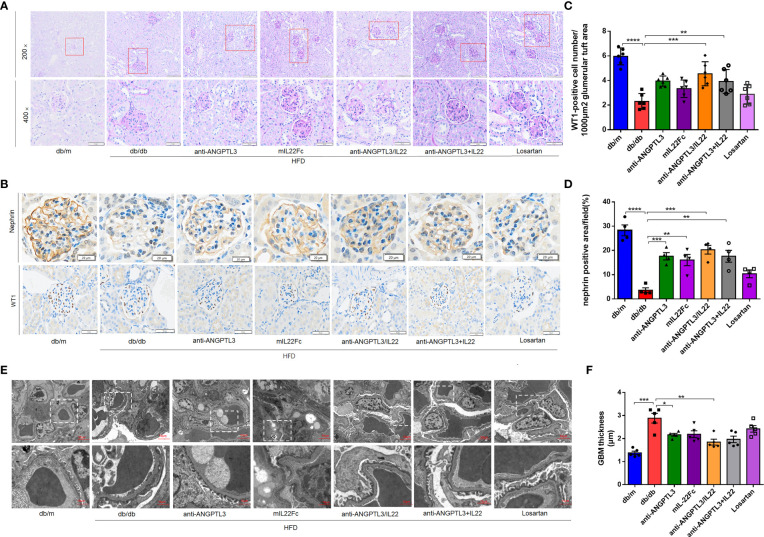
Treatment with anti-ANGPTL3/IL22 attenuated histological damage in DN mice **(A)** Representative images of PAS staining of kidney tissue from *db/db* mice. (Scale bar: 100 μm, 200 ×; 50 μm, 400 ×). **(B)** Expressions of nephrin and WT1 were detected by immunohistochemical staining. (Scale bar: 20 μm and 50 μm) **(C)** Quantification of WT1-positive cells in the glomerulus. **(D)** Statistical analysis of nephrin-positive areas in the kidney. **(E, F)** Representative electron microscopic images in *db/db* mice and quantitative analysis of glomerular basement membrane (GBM) thickness after the treatment of anti-ANGPTL3/IL22. (Scale bar: 10 μm) (n = 5-7). *P < 0.05; **P < 0.01; ***P < 0.001; ****P < 0.0001.

To analyze the effect of anti-ANGPTL3/IL22 on histological changes in DN, PAS staining was employed to show the degree of glomerular capsule adhesion and mesangial proliferation. Compared with *db/m* group, mice in *db/db* group displayed renal pathological changes in the absence of drug administration, including severe glomerular-capsular adhesions, increased mesangial matrix and capillary collaterals deformation (**Figure 4A**). The anti-ANGPTL3/IL22 fusion protein further alleviated the increased renal lesions compared with anti-ANGPTL3, mIL22Fc intervention alone and the combination group. However, Losartan exhibited only a slight improvement in renal histopathology.

Then the number of podocytes per glomerulus and the marker proteins of glomerular filtration barrier including WT1 and nephrin were investigated to verify glomerular function (**Figure 4B**). In comparison with *db/db* group, treatment with anti-ANGPTL3/IL22 showed more potent protection for podocytes, as revealed by the proportion of WT-1 positive podocytes in glomerular section, while anti-ANGPTL3 + IL22, mIL22Fc and Losartan groups showed weak protective efficiency (**Figure 4C**). Meanwhile, elevated levels of nephrin were shown in anti-ANGPTL3/IL22 intervention groups compared with the *db/db* group, indicating that the barrier function of glomerular filtration membrane was repaired (*P*<0.05) (**Figure 4D**). The diffuse uniform thickening of glomerular basement membrane (GBM), the earliest pathological change in DN, were also prominently reduced by the anti-ANGPTL3/IL22 compared with anti-ANGPTL3 or mIL22 alone (*P*<0.01). Both anti-ANGPTL3/IL22 and co-administration could reduced diffuse homogeneous thickening of the glomerular basement membrane (GBM) (**Figures 4E, F**).

### Anti-ANGPTL3-IL22 reduced renal collagen deposition and expression of fibrosis-related proteins

**Figure 5:**
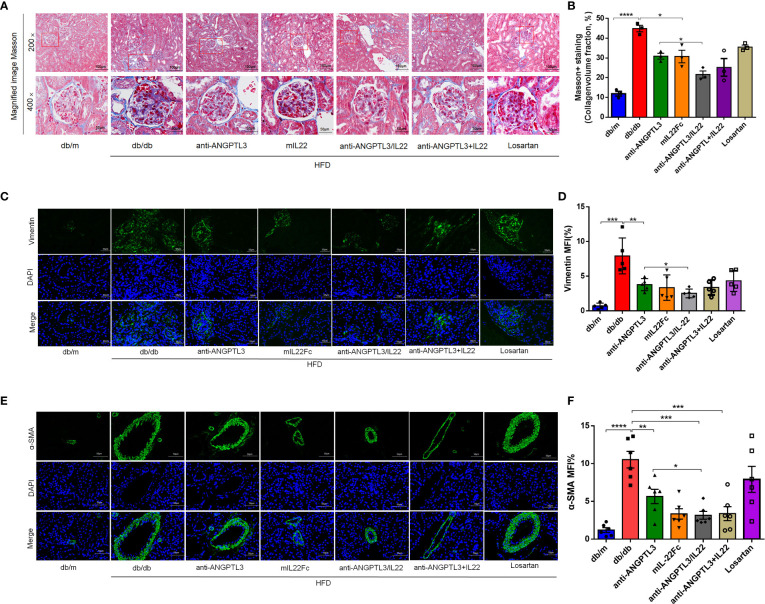
Anti-ANGPTL3/IL22 fusion protein reduced renal fibrosis in *db/db* mice. **(A)** Representative Masson staining images of mouse kidney tissues after 8 weeks of anti-ANGPTL3/IL22 treatment. **(B)** Measurements of Masson-positive staining. (Scale bar: 100 μm, 200 ×; 50 μm, 400 ×). **(C)** The levels of vimentin in the kidney sections were measured by immunofluorescence. (Scale bar: 50 μm). **(D)** Statistical assessment of vimentin expression in the kidney sections. (n = 5). **(E)** The levels of α-SMA in the kidney sections were measured by immunofluorescence. (Scale bar: 50 μm). **(F)** Quantitative analysis of α-SMA expression (n = 6). *P < 0.05; **P < 0.01; ***P < 0.001 ; ****P<0.0001.

During the progression of DN, the transformation of renal parenchymal cells to fibroblasts occurs under the stimulus of hyperglycemia, urinary protein and oxidative stress, which eventually induces renal fibrosis and ESRD (34, 35). To verify the extent of renal fibrosis remission, we assessed the intrarenal collagen accumulation by Masson staining and the expression of fibrosis-related proteins including vimentin and α-SMA in diabetic-induced renal tissues by immunofluorescence staining (**Figures 5A, C, E**). Quantitative analysis indicated that the anti-ANGPTL3/IL22 group attenuated renal collagen accumulation in the glomerular and tubular interstitium more significantly and altered the high expression of aforementioned fibrosis-related proteins compared to the anti-ANGPTL3, mIL22Fc and losartan groups (**Figures 5B, D, F**). Taken together, anti-ANGPTL3/IL22 significantly moderated kidney collagen accumulation and suppressed the expression of fibrosis-related proteins in DN, which might ultimately alleviate renal fibrosis.

3.6 Inhibitory efficacy of anti-ANGPTL3/IL22 fusion protein on NLRP3 inflammasome activation and inflammatory response

**Figure 6:**
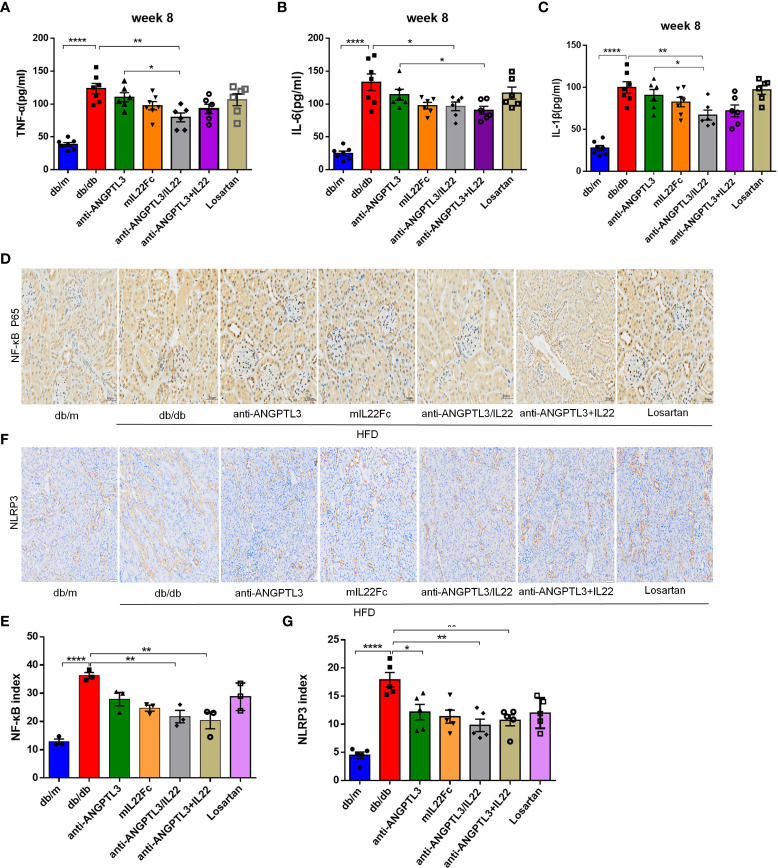
Anti-ANGPTL3/IL22 fusion protein attenuated the inflammatory response in *db/db* mice. **(A-C)** Measurements of serum inflammatory factors including TNF-α, IL-1β and IL-6 in *db/db* mice after treatment with anti-ANGPTL3/IL22. **(D)** Representative immunohistochemical images of NF-κB p65 in the kidney sections. (Scale bar: 20μm). **(E)** Representative immunohistochemical images of NLRP3 inflammasome in the kidney sections. (Scale bar: 50 μm). **(F)** Quantitative analysis of NF-κB. (n = 3). **(G)** Quantitative analysis of NLRP3 inflammasome (n = 5). *P < 0.05; **P < 0.01; ****P< 0.0001.

As shown in **Figures 6A–C**, diabetes induced the upregulation of serum TNF-α, IL-6, and IL-1β, and treatment with anti-ANGPTL3/IL22 showed prominent reversal compared with mIL22Fc or anti-ANGPTL3 alone. High glucose could induce NF-κB phosphorylation and subsequently trigger transcriptional activation of NLRP3 inflammasome, which was proven to increase in DN and contribute to renal injury (36–38). In this research, anti-ANGPTL3/IL22 significantly inhibited NLRP3 inflammasome and NF-κB p65 activation compared with anti-ANGPTL3 or mIL22Fc alone, which was similar to the anti-ANGPTL3 + IL22 group (**Figures 6D–G**). In summary, anti-ANGPTL3/IL22 could attenuate NLRP3 activation and inhibit inflammatory responses more effectively, thereby protecting renal function in DN mice.

### Inflammatory response and metabolism-related processes were involved in transcriptomic changes following anti-ANGPTL3/IL22 administration

**Figure 7:**
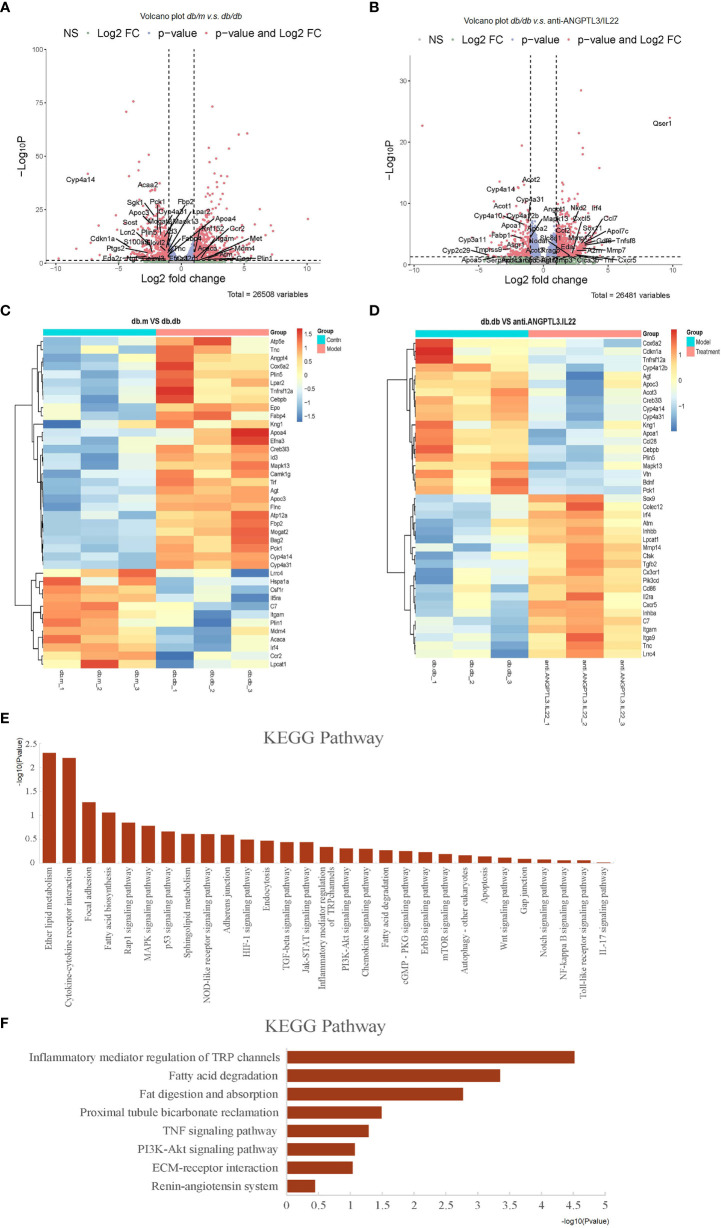
Transcriptomic analysis of *db/db* mice treated with anti-ANGPTL3/IL22. **(A)** Differential expression of genes related to inflammation, podocyte injury and metabolism in *db/m* mice compared to *db/db* mice were shown in volcano plots (log2 fold change ≥ 1 or ≤ -1 in the horizontal coordinate and p value ≤ 0.05 in the vertical coordinate). **(B)** Differential expression of genes related to inflammation, podocyte injury and metabolism (log2 fold change ≥ 1 or ≤ -1 in the horizontal coordinate and p value ≤ 0.05 in the vertical coordinate) in *db/db* compared to anti-ANGPTL3/IL22 treatment mice. **(C)** Cluster analysis of differentially expressed genes including inflammation-related, metabolism-related and podocyte damage process-related genes between *db/m* and *db/db* mice. **(D)** Cluster analysis of differentially expressed genes including inflammation-related, metabolism-related and podocyte damage process-related genes between anti-ANGPTL3/IL22 treated mice and *db/db* mice. **(E)** KEGG analysis of genes significantly up-regulated in *db/db* mice. **(F)** KEGG analysis of genes obviously down-regulated in anti-ANGPTL3/IL22 administration mice.

To better explore the possible mechanisms associated with the improvement of DN by anti-ANGPTL3/IL22, transcriptome sequencing was performed on kidney tissues. As shown in the volcano plot, compared with the *db/m* group, a total of 2195 genes were markedly altered in *db/db* group (**Figures 7A, B**). Cluster analysis revealed that differentially expressed genes in DN were mainly involved in the processes of renal podocyte injury (e.g., *Itgam*and *Plin5*), glucolipid metabolism (e.g., *Cyp4a14*and *Cyp4a31*) and inflammatory response (e.g., *Mapk13*and *Tnfrsf12a*), which were altered after treatment with anti-ANGPTL3/IL22 (**Figures 7C, D**).

According to Kyoto Encyclopedia of Genes and Genomes (KEGG) analysis, signaling pathways that were significantly downregulated after anti-ANGPTL3/IL22 administration mainly involved inflammatory mediator regulation (KEGG: mmu04750), TNF signaling pathway (KEGG: mmu04668), fatty acid metabolism-related pathway (KEGG: mmu00071, KEGG: mmu04975), PI3K-related pathway (KEGG: mmu04151), ECM-receptor interaction (KEGG: mmu04512), etc., suggesting that anti-ANGPTL3/IL22 may promote the repair of renal function by alleviating podocyte injury, reducing excessive inflammatory response leading to collagen deposition, and regulating metabolic disorders (**Figures 7E, F**).

## Discussion

DN is a severe microvascular complication of diabetes that occurs in up to 50% of people with diabetes. DN is a common cause of ESRD, which eventually requires replacement therapy such as dialysis and kidney transplantation, imposing a considerable burden on the individuals and socioeconomic costs (2, 39). Extensive data point to uninterrupted albuminuria and inflammatory response as key factors in the pathogenesis of DN (19, 40). Therefore, the emergence of new approaches for protection of podocytes and the suppression of inflammatory responses may offer further opportunities for DN therapy.

Herein, we provided evidence for the application of anti-ANGPTL3/IL22 fusion protein as an effective treatment for DN. Anti-ANGPTL3/IL22 fusion protein was expressed, purified and then examined for its protective efficacy and the possible mechanism in the *db/db* murine model of type 2 diabetes. Our results indicated that anti-ANGPTL3/IL22 administration attenuated podocyte injury and ACR, while ameliorating renal fibrosis and regulating glucolipid metabolism by suppressing the inflammatory response.

Loss of podocyte function leads to the proliferation of mesangial cells, accumulation of ECM, and denudation of GBM, whereas greater damage cause glomerulosclerosis and interstitial fibrosis (41, 42). Reducing proteinuria may delay the progression of DN by improving the functions of podocytes. In our previous studies, elevated ANGPTL3 was positively correlated with podocyte injury and proteinuria formation through the integrin αVβ3/FAK/PI3K signaling pathways (16, 43). We also demonstrated that monoclonal antibody targeting ANGPTL3-FLD was effective in alleviating podocyte injury and protect renal function in the adriamycin-induced renal injury model. Consistent with the previous reports, the present study found that the detrimental changes such as massive proteinuria, high BUN, low WT1, and nephrin expression in renal parenchymal cells in *db/db* group were improved by anti-ANGPTL3 antibody, mIL22Fc and anti-ANGPTL3/IL22 compared with *db/db* group. In particular, anti-ANGPTL3/IL22 fusion protein obtained the best therapeutic effect against DN. Eight weeks of anti-ANGPTL3/IL22 treatment not only contributed to a sustained reduction in albuminuria, but also moderated histological deterioration including pedunculated exudate and thylakoid expansion. The results of PAS staining and EM revealed that anti-ANGPTL3/IL22 fusion protein improved glomerular mesangial matrix hyperplasia, morphology of intra-glomerular capillary collaterals and foot process effacement, showing the strongest protective effect against DN in all groups. Furthermore, anti-ANGPTL3/IL22 fusion protein could efficiently elevate renal index under high glucose conditions through alleviating renal tubular and renal interstitial damage, while only marginal improvements were achieved in the anti-ANGPTL3, mIL22Fc and losartan intervention group.

Inflammation has been proven by pathological examinations and epidemiological investigations to be a major factor in the pathogenesis of DN (20). Pro-inflammatory cytokines decreased GFR and affect the proliferation of mesangial cells (44, 45). Moreover, the activation of NF-κB/NLRP3 is closely associated with certain chemokines, cell adhesion proteins and pro-inflammatory cytokines, further resulting in the progression of DN (38, 46). IL22 has been demonstrated to reduce ROS production and ameliorate inflammation by downregulating the NF-κB/NLRP3 signaling pathway, thereby promoting regeneration and repair of renal epithelial tissue (33, 47). In the current study, we found that anti-ANGPTL3/IL22 could effectively reduce the production of inflammatory factors including IL-6, TNF-α and IL-1β by inhibiting NF-κB p65/NLRP3 inflammasome, suggesting that the beneficial effect of anti-ANGPTL3/IL22 in the treatment of DN may be attributed to the attenuation of the inflammatory response. In terms of podocyte function, anti-ANGPTL3/IL22 better improved urinary ACR and the expressions of WT1 and nephrin in renal tissues compared to anti-ANGPTL3 antibody alone. Hitherto, no studies have identified the efficacy of anti-ANGPTL3 in regulating inflammation. However, we found for the first time that blocking ANGPTL3 signaling attenuated NF-κB p65/NLRP3 inflammasome activation and shortened IL-1β production to a lesser extent. The relationship between ANGPTL3 activation and inflammation needs to be further explored. Taken together, the favorable outcome of anti-ANGPTL3/IL22 on DN is possibly associated with the mitigation of inflammatory response involving NF-κB and NLRP3.

Podocyte injury is related to inflammation and occupies a crucial place in renal fibrosis. In brief, proteinuria induces activation of renal tubular cells and subsequently leads to increased expressions of chemokines, adhesion molecules, and pro-inflammatory cytokines (48, 49). Circulating monocytes, neutrophils and lymphocytes are then recruited into the renal tissue, which impairs the glomerular filtration membrane and tubular interstitium, ultimately contributing to glomerulosclerosis and interstitial fibrosis and further exacerbating proteinuria (21, 22). In our study, the application of anti-ANGPTL3/IL22 was more effective in reducing collagen deposition due to remission of proteinuria and inflammatory cytokines compared to treatment with anti-ANGPTL3 or mIL22Fc alone. Also, the expression of fibrosis-associated proteins vimentin and α-SMA in renal tissues was significantly decreased after anti-ANGPTL3/IL22 treatment, suggesting that the fusion proteins could mitigate the progression of renal fibrosis by alleviating podocyte injury and inhibiting inflammatory responses.

In some instances, comparisons of parameters between anti-ANGPTL3/IL22 and anti-ANGPTL3 + IL22 treatments showed little difference. Either anti-ANGPTL3/IL22 or co-administration significantly ameliorated kidney dysfunction and reduced diffuse homogeneous thickening of the glomerular basement membrane (GBM). The anti-ANGPTL3/IL22 fusion protein group inhibited NLRP3 inflammasome and suppressed inflammatory responses, which was similar to the anti-ANGPTL3 + IL22 group. However, it is worth noting that the bifunctional fusion protein was more effective in inhibiting renal fibrosis and protecting podocytes compared with the combination group. As shown in PAS staining, the degree of glomerular capsule adhesions was alleviated to a greater extent in the anti-ANGPTL3/IL22 fusion protein group in comparison with the combination group. In addition to this, treatment with anti-ANGPTL3/IL22 showed more potent protection of podocytes, as revealed by the proportion of WT-1 and nephrin-positive podocytes in the glomerular sections. Finally, the anti-ANGPTL3/IL22 fusion protein prolonged the half-life of IL22, and is expected to reduce the production costs compared to producing two proteins separately. Taken together, administration of anti-ANGPTL3/IL22 fusion protein may be a more desirable therapeutic strategy than co-administration of anti-ANGPTL3 and IL-22 in the treatment of DN.

In addition to renal interstitial fibrosis and glomerulosclerosis, the disturbance of glucose and lipid metabolism is considered to be a critical factor in the development and progression of DN (50, 51). Abnormally elevated blood glucose and lipid deposition can induce podocyte death, renal tubular interstitial fibrosis and insulin resistance (52). Thus, maintaining the endocrine environment and metabolic homeostasis becomes an urgent issue in preventing DN from progressing to ESRD. IL-22 has been reported to effectively improve insulin resistance and lipid metabolism disorders in the therapy of DN (32). Similarly, in our study, anti-ANGPTL3/IL22 fusion protein has a more dramatic effect in lowering blood glucose and abnormal lipid (including T-CHO, LDL cholesterol, and triglycerides) than treatment with anti-ANGPTL3 and mIL22Fc treatment alone. Moreover, disorders of glucose and lipid metabolism and inflammatory responses are mutually reinforcing in the pathogenesis of DN. Abnormalities in glucose and lipid metabolism can trigger a range of cytokines that mediate and amplify inflammatory response and oxidative stress (53). In summary, our findings suggest that anti-ANGPTL3/IL22 fusion protein protects the kidney by reducing proteinuria and renal fibrosis as well as stabilizing the endocrine environment in the treatment of DN.

## Conclusion

Proteinuria, inflammatory response, and metabolic disturbance are risk factors for the progression of DN to ESRD. Our study shows that anti-ANGPTL3/IL22 ameliorates DN by attenuating renal injury, inflammation and dysfunctional glucolipid metabolism. Mechanistically, the nephroprotective effect of anti-ANGPTL3/IL22 on DN is due to the blockade of NF-κB/NLRP3 pathway. The application of anti-ANGPTL3/IL22 may be a hopeful therapeutic approach for the treatment of DN.

## Data availability statement

The original contributions presented in the study are publicly available. The data presented in the study are deposited in the NCBI SRA repository. The accession number is PRJNA901225.

## Ethics statement

The animal study was reviewed and approved by Ethics Committee of School of Pharmacy, Fudan University.

## Author contributions

HX, DJ and HH instructed and revised the manuscript. QM, XH, FL, ZC and LH carried out experiments. KZ, YB, YZ, YN and QL analysis the data; FL, JR, XY and TW made the figures; QM, XH and FL drafted and revised the paper. All authors have read and approved the final manuscript. The final version of the paper has been approved by all authors.

## Funding

This research was funded by the National Natural Science Foundation of China under grant 82170793 and The Collaborative Research Projects of Greater Bay Area Institute of Precision Medicine (Guangzhou) in 2021 (IPM2021C003).

## Conflict of interest

The authors declare that the research was conducted in the absence of any commercial or financial relationships that could be construed as a potential conflict of interest.

## Publisher’s note

All claims expressed in this article are solely those of the authors and do not necessarily represent those of their affiliated organizations, or those of the publisher, the editors and the reviewers. Any product that may be evaluated in this article, or claim that may be made by its manufacturer, is not guaranteed or endorsed by the publisher.

## Abbreviations

DN, Diabetic nephropathy; ESRD, end-stage renal disease; ANGPTL3, angiopoietin-like protein 3; IL-22, Interleukin-22; NLRP3, NOD-like receptor family pyrin domain-containing protein 3; NF-κB, nuclear factor-kappa lightchain enhancer of activated B cells; CRF, chronic renal failure; GFR, glomerular filtration rate; CCD, N-terminal coiled-coil domain; FLD, C-terminal fibrinogen-like domain; LPL, lipoprotein lipase; NS, nephrotic syndrome; ACR, urine albumin-to-creatinine ratio; BUN, blood urea nitrogen; Ccr, creatinine clearance rate; ECM, extracellular matrix; GBM, glomerular basement membrane; H&E, hematoxylin & eosin; KEGG, Kyoto Encyclopedia of Genes and Genomes; PAS, periodic acid-Schiff; ROS, reactive oxygen species; SDS-PAGE, SDS-polyacrylamide gel electrophoresis; SPR, Surface Plasmon Resonance; MPC5, mouse glomerular podocytes; mPTC, mouse proximal renal tubular epithelial cells; ECM, extracellular matrix.
