# Supplementary material for: A novel fusion protein consisting of anti-ANGPTL3 antibody and interleukin-22 ameliorates diabetic nephropathy in mice
Source: Front Immunol. 2022 Dec 5;13:1011442. doi: 10.3389/fimmu.2022.1011442 (PMC9760875; doi:10.3389/fimmu.2022.1011442)
Supplement: Supplementary file 1 [file DataSheet_1.docx]

Supplementary Material

## Supplementary Figures 1


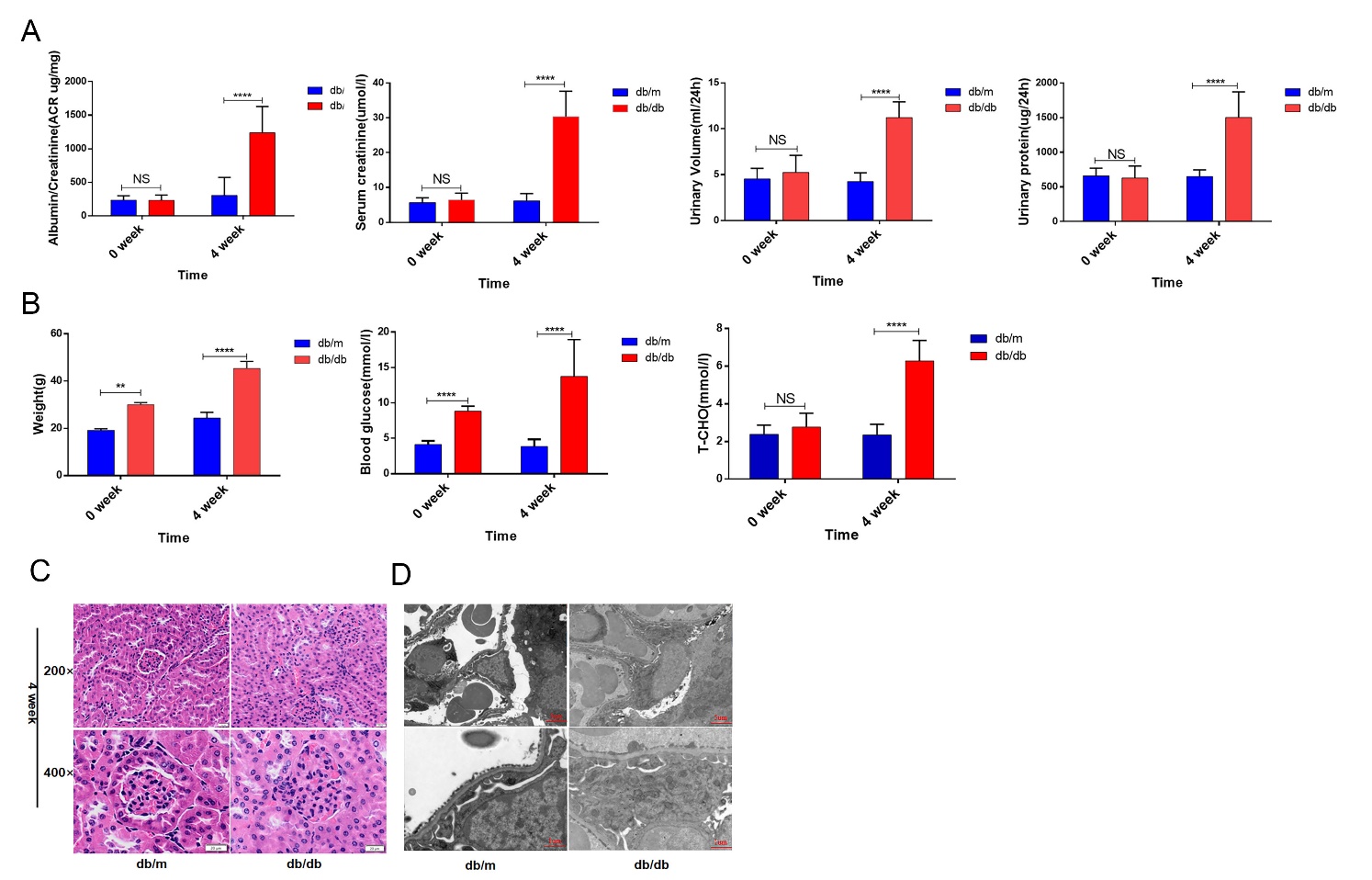


**Supplementary Figure 1.** (A) Measurements of ACR, serum creatinine, and 24-hour urine volume and urine protein. (B) Measurements of body weight, blood glucose and total cholesterol (T-CHO) (C) H&E staining of kidney tissues of *db/m* mice and *db/db* mice at week 4 of the experiment. (Scale bar: 50 μm, 200 ×; 20 μm, 400 ×). (D) Electron microscopy images of *db/m* and *db/db* mice at week 4 of the experiment. Figure (A) - (D) showed that the mouse model of DN was successfully constructed as evidenced by mouse serological examination, kidney tissue and electron microscopic ultrastructure (Scale bar: 5 μm) (n = 5). **P* < 0.05;***P* < 0.01; ****P* < 0.001.

## Supplementary Figures 2


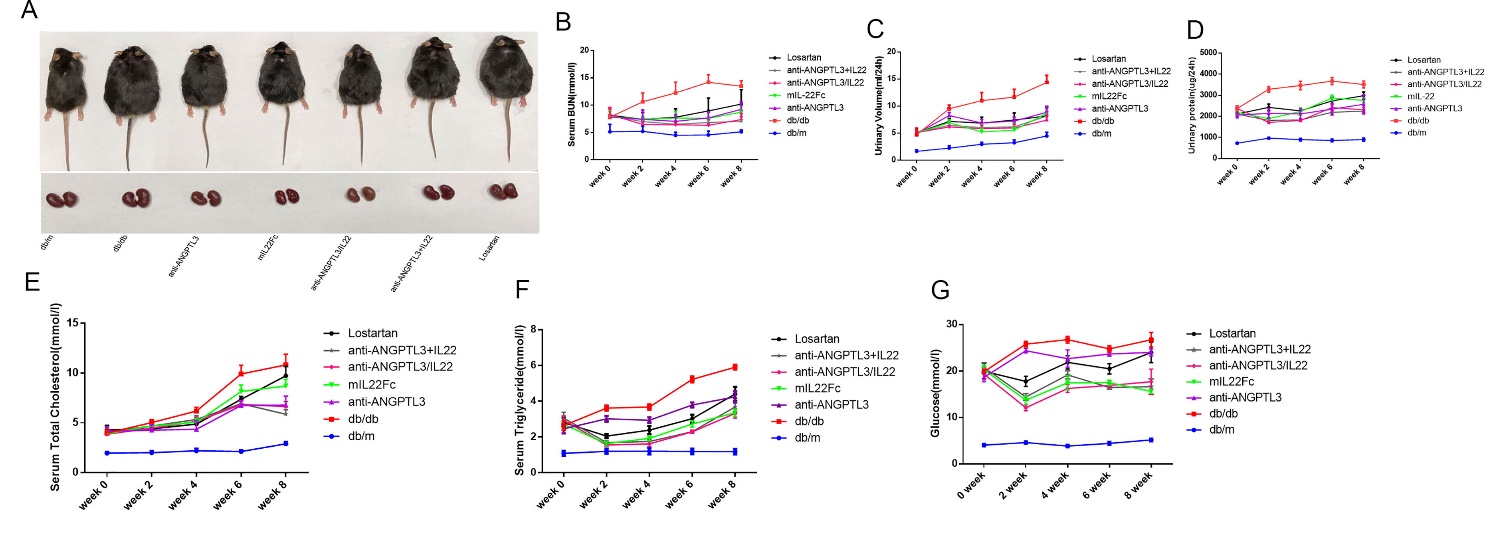


**Supplementary Figure 2.** (A) Measurements of body weight and renal weight at twelve weeks. (B) - (D) The tendency of serum BUN, 24-hour urine volume and urine protein during eight weeks of drug administration. (E) - (G) Measurements of serum triglyceride, total cholesterol and blood glucose.

## Supplementary Figures 3


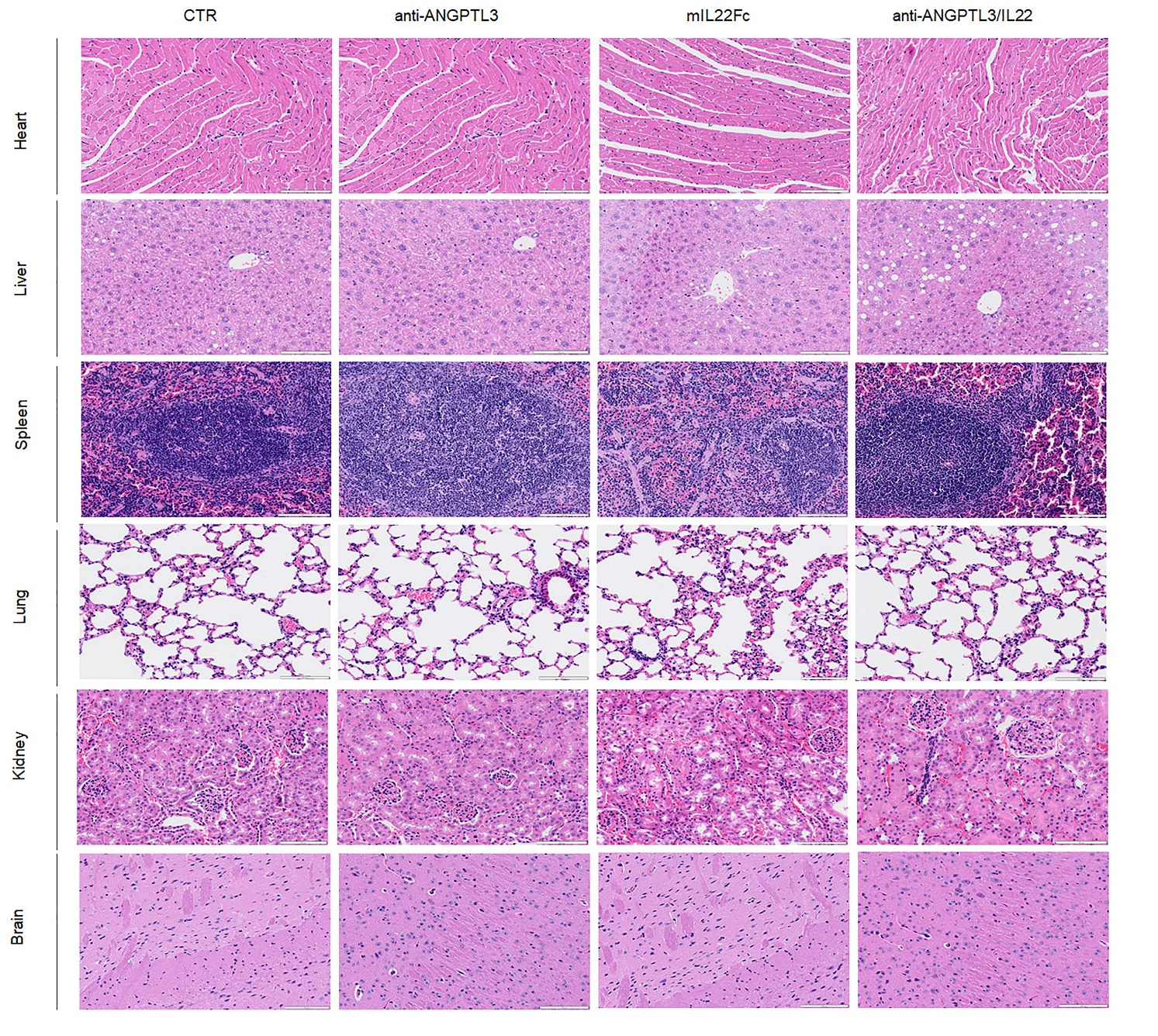


**Supplementary Figure3.** Histological examination of major organs after treatment with anti-ANGPTL3/IL22, anti-ANGPTL3 and mIL22Fc.
